# Orthodontics in Pediatric Osteoporosis: A Narrative Literature Review

**DOI:** 10.3390/children12060691

**Published:** 2025-05-28

**Authors:** Anastasia Panagiota Gravia, Heleni Vastardis, Apostolos I. Tsolakis, Artemis Doulgeraki

**Affiliations:** 1 Metabolic Bone Diseases, School of Medicine, National and Kapodistrian University of Athens, 14561 Athens, Greece; 2 Department of Orthodontics, School of Dentistry, National and Kapodistrian University of Athens, 11527 Athens, Greece; vastard@dent.uoa.gr (H.V.); atsolaki@dent.uoa.gr (A.I.T.); 3 Department of Orthodontics, Case Western Reserve University, Cleveland, OH 44106, USA; 4 Department of Bone and Mineral Metabolism, Institute of Child Health, 11527 Athens, Greece; doulgeraki@ich.gr

**Keywords:** pediatric osteoporosis, orthodontics, osteogenesis imperfecta, bisphosphonates, malocclusion

## Abstract

Osteoporosis is the most common metabolic bone disorder, characterized by reduced bone mass and abnormal bone microarchitecture, resulting in increased bone fragility and a heightened risk of low-energy fractures. Pediatric osteoporosis may be either primary, due to genetic factors, or secondary, arising from chronic diseases and/or their treatment. Oral health and proper occlusion are integral components of overall health, influencing functionality, nutrition, facial aesthetics, and psychosocial development during childhood. Severe malocclusion can adversely affect speech, mastication, appearance, psychological well-being, and social interactions. The aim of this narrative review is to examine the existing literature on orthodontic anomalies and management strategies in pediatric patients with osteoporosis while highlighting clinical challenges, treatment limitations, and areas necessitating further research. A comprehensive literature search was conducted in the PubMed database, focusing on studies involving human subjects aged 3 to 18 years, published in English between 2002 and 2024. The findings indicate that children with osteoporosis present with more severe dental and occlusal complications compared to their healthy peers, often facing increased orthodontic complexity due to skeletal fragility and systemic comorbidities. These challenges necessitate careful, individualized treatment planning and close multidisciplinary collaboration. Although research in this field remains limited due to the rarity of pediatric osteoporosis, recognizing and addressing the specific needs of this population is critical to improving clinical outcomes and guiding future therapeutic approaches.

## 1. Introduction

Osteoporosis is the most prevalent metabolic bone disorder, defined by low bone mass and the deterioration of bone microarchitecture, leading to reduced bone strength and a heightened risk of fractures from minimal impact [1]. In the past, osteoporosis was regarded as a disease predominantly affecting the elderly. However, in recent decades, it has been increasingly diagnosed in children. In pediatric cases, clinical features, diagnostic criteria, monitoring protocols, and treatment strategies differ substantially from those in adults. Pediatric osteoporosis may occur either due to genetic disorders affecting the bones (primary pediatric osteoporosis) or, more commonly, due to chronic diseases or medical treatments affecting the skeleton (secondary pediatric osteoporosis) [1,2,3,4].

Primary osteoporosis in children refers to rare, genetically determined conditions that impair bone development and structure, resulting in low bone mass and increased fragility without any identifiable underlying disease [2]. The most prevalent and well-characterized form of primary osteoporosis is osteogenesis imperfecta (OI), a hereditary connective tissue disorder caused primarily by mutations in collagen-related genes. OI is marked by frequent fractures, bone deformities, and other systemic features, and affects approximately 1 in 20,000 live births [1,2,3,4]. In contrast, secondary pediatric osteoporosis arises as a consequence of chronic systemic illnesses or their treatment. In such cases, bone fragility results from a multifactorial disruption of bone metabolism, including inflammation, hormonal imbalances, reduced mobility, and the side effects of medications such as glucocorticoids [1,2].

The diagnosis of pediatric osteoporosis requires a comprehensive medical history, including family and dietary history, a thorough clinical examination with growth curve plotting, and a complete laboratory and radiological workup [1,2,4,5].

The revised 2019 Pediatric Positions Diagnostic Criteria of the International Society of Clinical Densitometry (ISCD) define pediatric osteoporosis as the presence of at least one unexplained vertebral fracture, even in the absence of low bone mineral density; or at least two low-energy fractures in children younger than 10 years; or at least three pathological fractures in children aged 10 to 18 years, in conjunction with a low bone mineral density (defined as a Z-score below −2.0 relative to age-and sex-matched healthy controls, corrected for body size, when indicated). Fractures of the skull, sternum, ribs, and phalanges are excluded from these criteria [1,4,5].

A key clinical characteristic of this disorder is reduced bone strength, which predisposes affected individuals to pathological fractures during childhood (fragility fractures or low-energy fractures). These fractures are further classified based on their location into vertebral and long bone fractures [1]. Long bone fractures are typically symptomatic, whereas vertebral fractures may remain undiagnosed due to subtle or absent clinical manifestations, particularly in cases of low severity or in patients with a history of chronic corticosteroid therapy, which may mask skeletal pain. The incidental detection of non-traumatic vertebral fractures (VFs) on lateral spine radiographs can also lead to the diagnosis of osteoporosis, irrespective of bone mineral density values [1,4,6].

During childhood and adolescence, up to 95% of total bone and muscle mass is acquired; therefore, this period is considered critical for the development of a robust musculoskeletal system [1]. The primary determinants of bone structure and quality include genetic predisposition, pharmacological interventions, neuromuscular and metabolic disorders, nutritional status, and chronic systemic diseases. Any disruption in this dynamic bone-building process may lead to an increased risk of fractures, not only during childhood but also later in adulthood [3,4,6].

In children and adolescents with osteoporosis, early diagnosis is crucial, as it facilitates the timely initiation of appropriate treatment and helps prevent severe complications. These complications include failure to achieve peak bone mass, skeletal deformities, growth retardation, and a loss of musculoskeletal function, all of which negatively impact quality of life and contribute to long-term health consequences [2,3,4,6].

The management of pediatric osteoporosis involves a combination of lifestyle modifications, treatment of the underlying cause, and pharmacological interventions. Lifestyle measures include an adequate dietary intake of calcium and vitamin D, regular exercise, and avoidance of osteotoxic drugs in cases of secondary osteoporosis [1,3,4]. Pharmacological treatment, particularly the off-label use of bisphosphonates, is only considered in selected cases, such as children with a high number of fractures, severe skeletal deformities, or persistent bone pain [1]. Bisphosphonates act by suppressing bone metabolism, primarily through the inhibition of bone resorption. Among them, pamidronate and zoledronate are the preferred agents for the treatment of moderate to severe osteoporosis in children [1,2,4,7], while denosumab is currently regarded as a second-line therapy, with only a limited number of reported cases in the literature [7].

Dental health and proper occlusion are integral components of overall health. Orthodontics, as a specialty of dentistry, focuses on the study of facial growth and development, the alignment of teeth within the dental arches, the relationship between the dental arches and jawbones, and the function of occlusion. It also encompasses the diagnosis, prevention, and treatment of malocclusion [8]. Orthodontic treatment is essential, as abnormalities in occlusion can negatively impact oral health, predisposing individuals to dental caries, periodontal disease, and occlusal trauma, as well as the function of the temporomandibular joint (TMJ), speech, mastication, and nutrition. Additionally, malocclusion can influence both well-being and social behavior [8,9,10].

The aim of this narrative review is twofold: (a) to examine the available literature regarding orthodontic anomalies in children and adolescents with pediatric osteoporosis, with particular emphasis on the impact of osteoporosis and its pharmacological management with bisphosphonates on occlusion and craniofacial development; and (b) to highlight the clinical challenges, treatment limitations, and areas requiring further research in the orthodontic management of this patient population.

## 2. Materials and Methods

The literature search was conducted in the PubMed electronic database using the Boolean operators AND/OR to combine the following terms: “orthodontic treatment”, “malocclusion”, “dentition”, and one keyword from each of the following categories: “pediatric osteoporosis”, “osteogenesis imperfecta”, “Bruck syndrome”, “Cole-Carpenter syndrome”, “bisphosphonates”, “denosumab”, “secondary osteoporosis”, “Ehlers-Danlos syndrome”, “cerebral palsy”, and “Duchenne muscular dystrophy”. Articles included in this review were studies that integrated information from both the field of orthodontics and pediatric osteoporosis. The search was restricted to studies conducted on human subjects, aged 3 to 18 years, and written in English. The literature search was also conducted using the PubMed database, covering the period from January 2002 to December 2024. Although only the PubMed database was used, it was selected due to its comprehensive coverage of biomedical and dental journals, ensuring high relevance to the topics of pediatric osteoporosis and orthodontics.

## 3. Results

A total of 386 articles were retrieved from the PubMed electronic database. Duplicate publications were excluded using Zotero reference management software (version 7.0.15), resulting in 320 unique records. These records were screened for eligibility based on their titles and abstracts. Studies were excluded if they focused solely on adult populations, addressed osteoporosis unrelated to orthodontic management, lacked orthodontic or dental outcomes, or were purely molecular/basic science studies without clinical applicability. Additionally, review articles, editorials, and conference abstracts were also excluded. Following this initial screening, 85 publications met the criteria for full-text review. During this stage, an additional 35 studies were excluded because the full text could not be retrieved or because they did not meet the inclusion criteria. Ultimately, 50 studies were considered eligible and included in the final analysis (references).

## 4. Primary Osteoporosis and Dentofacial Orthopedics

Primary (genetic or early-onset) osteoporosis refers to hereditary bone fragility disorders characterized by abnormal bone tissue synthesis. It is highly heterogeneous, encompassing mild to life-threatening forms, resulting in a broad spectrum of skeletal and non-skeletal manifestations in affected children. The underlying genetic mutations influence multiple aspects of bone metabolism, including collagen type I synthesis, bone mineralization, and the function of osteoblasts and osteoclasts, ultimately leading to bone disease [1,2,3,4]. Intervention with appliances and techniques of orthodontics and dentofacial orthopedics in order to move teeth and redirect mandibulofacial growth should be performed at an early age since the patient’s dentition and malocclusion tend to worsen over time [11].

### 4.1. Osteogenesis Imperfecta: Orofacial Manifestations and Orthodontic Treatment

Osteogenesis imperfecta (OI) is the most common and extensively studied form of primary osteoporosis. However, it remains a rare disease, with an estimated prevalence of 1 in 20,000 births. Its pathophysiology involves defects in the synthesis or structural organization of collagen, the primary component of bone matrix. The hallmark feature of OI is an increased susceptibility to low-energy fractures. The most common types of OI follow an autosomal dominant inheritance pattern, whereas rarer forms exhibit autosomal recessive or X-linked inheritance. To date, mutations in more than 24 genes have been identified as causative factors for this disease. In 90% of cases, OI results from mutations in COL1A1 or COL1A2, the genes encoding the α1 and α2 chains of collagen type I. In other cases, mutations typically affect genes involved in the post-translational modification of collagen type I. The clinical spectrum of OI varies widely, ranging from mild phenotypes to severe forms that may result in perinatal or neonatal lethality [12,13,14,15]. The revised Sillence clinical classification describes five types of OI, each of which is associated with one or more causative genes [16]. Type I is the mildest form, type II is lethal, type III is the most severe non-lethal form, and types IV and V present moderate severity. The primary clinical features of OI appear at different stages of life, depending on the underlying genetic mutation and the severity of the phenotype. These include recurrent fractures, skeletal deformities, dentinogenesis imperfecta (DGI), short stature, blue sclerae, hearing deficits, and ligamentous laxity [1,2,3,4,6]. Existing evidence indicates that individuals diagnosed with OI are more likely to present with complex and distinct malocclusion patterns compared to those without the condition [11,12,17].

The teeth of individuals with OI exhibit a reduced mesiodistal dimension compared to healthy controls. The magnitude of this reduction varies by tooth type, with a mean decrease of 0.17 mm. Incisors and first premolars are the most commonly affected, whereas molars exhibit the least reduction in size. When OI coexists with DGI, the impact on dentin collagen is more pronounced, both qualitatively and quantitatively, resulting in a greater reduction in mesiodistal tooth dimensions compared to individuals with OI alone. The effect of OI on mesiodistal tooth dimensions appears to be independent of sex and jaw location. This reduction in tooth size partially explains the less pronounced dental crowding observed in individuals with OI compared to their healthy peers [18].

In addition to congenital deficiencies, unerupted teeth are frequently observed in children with OI. This phenomenon is particularly evident in second permanent molars, primarily in the maxilla, where the incidence of delayed eruption or complete failure of eruption is remarkably high [OI type III (70%), OI IV (35%), and OI I (2%)], an occurrence that is exceedingly rare in healthy individuals (0–2.3%) [19,20]. Notably, these unerupted teeth do not appear to be associated with any physical obstruction in their eruption pathway. The majority of these unerupted molars were located in the maxilla. According to Andersson et al., a possible explanation for this phenomenon is the craniofacial growth pattern in OI, which may negatively influence the eruption of second permanent molars. In contrast, bisphosphonate therapy (discussed in detail below) does not appear to affect the eruption of these teeth [19].

Another significant challenge for orthodontists treating patients with OI is the increased prevalence of dental agenesis. According to Taqi et al., individuals with OI have an average of 2.4 congenitally missing teeth and 0.8 unerupted teeth per patient. The prevalence of congenitally missing or unerupted teeth is notably higher in individuals with OI and varies depending on tooth type and OI subtype, with the highest rates observed in types III, IV, and I. Specifically, patients with moderate to severe OI had a considerably greater prevalence of tooth agenesis, with 52% of those with OI type IV and 61% of those with OI type III affected, compared to only 11% of patients with OI type I, while in the general population, the average proportion is about 6.5%. This study also found that patients with OI most commonly lack first premolars, canines, first molars, and central incisors. The high prevalence of dental agenesis in these individuals may result from the deleterious effects of structurally abnormal type I collagen on tooth germ formation and/or the early stages of tooth development and mineralization. Anterior teeth are more frequently missing in the maxilla, whereas posterior teeth are more commonly absent in the mandible. These findings may be associated with the underdeveloped maxilla and overdeveloped mandible typically observed in OI patients, particularly in types III and IV [20].

Malmgren et al. also report that dental agenesis is common in children with OI, with a prevalence of approximately 17%. According to this study, the incorporation of structurally abnormal collagen may disrupt interactions with the extracellular matrix surrounding the developing tooth, leading to impaired mineralization and the inhibition of early tooth formation, ultimately resulting in dental agenesis [21].

In the general population, orthodontic anomalies associated with Class II skeletal patterns are approximately three times more prevalent than those related to Class III patterns. However, children and adolescents with OI predominantly exhibit Class III skeletal anomalies. The mandible in these individuals tends to appear more prominent and asymmetric [13,19,22]. This skeletal discrepancy is primarily attributed to maxillary growth retardation and hypoplasia, combined with a tendency for mandibular hyperplasia, which has been associated with a single-nucleotide mutation in the COL1A1 gene [15]. OI patients exhibit midface hypoplasia along with mandibular hyperplasia and concurrent facial asymmetry, particularly in the lower third of the face [13].

Numerous studies have demonstrated the high prevalence and severity of anterior and posterior crossbite in individuals with OI. This can be attributed to the increased incidence of dentoskeletal Class III malocclusion, which is strongly associated with bilateral posterior crossbite. Similarly, the lower prevalence of increased horizontal and dental vertical overlap in children with OI compared to their healthy peers can be explained by the strong correlation with Class II malocclusion, which is rarely observed in OI patients [11,12]. A study by Rizkallah et al., involving 49 patients aged 5 to 19 years with OI, confirmed that Class III malocclusion was significantly more frequent and severe than in the control group. Additionally, anterior and posterior open bite, anterior crossbite, and craniofacial deformities—including a triangular facial shape and protuberance of the temporal and frontal bones—were among the most notable findings [12].

Patients with type 1 OI and mild DGI who present with moderate malocclusions may be successfully managed using conventional orthodontic techniques [11,23]. For example, light intermaxillary elastics are often a more suitable option for anteroposterior correction, as they apply less mechanical stress to the dentition and supporting skeletal structures compared to traditional protraction appliances [24].

Oromandibular dysfunction is commonly observed in children with OI and encompasses chewing and swallowing difficulties, hypersensitivity of the masticatory muscles, excessive drooling, and temporomandibular joint (TMJ) disorders, including joint hypermobility, mandibular mobility restrictions, and subluxation. Additionally, OI is frequently characterized by DGI, as well as other dental anomalies, such as tooth agenesis and occlusal irregularities [25].

**Figure 1:**
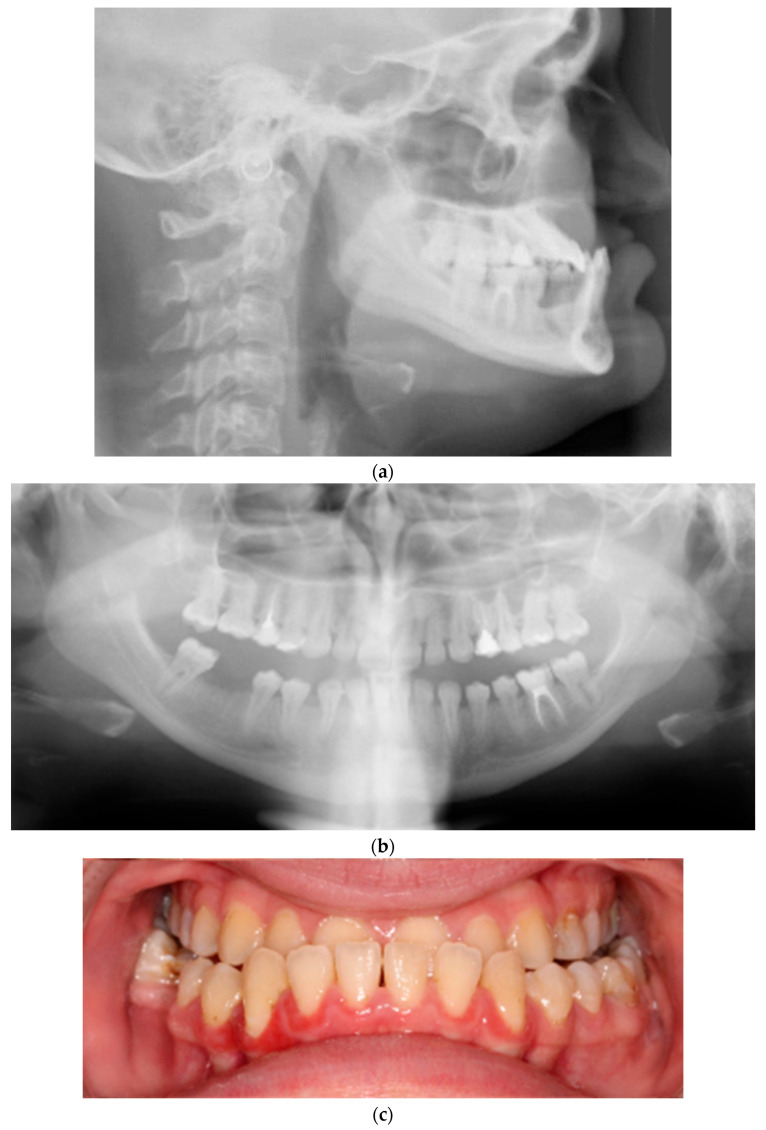
(**a**) Lateral cephalogram, (**b**) panoramic radiograph, and (**c**) frontal intraoral photograph of a 16-year-old female patient with OI. The patient presents a Class III malocclusion, prognathism, and anterior and posterior crossbite.

In summary, OI in children and adolescents is frequently associated with a spectrum of dental and craniofacial anomalies, including DGI, tooth agenesis, a delayed eruption of second permanent molars, anterior or posterior crossbite, prognathism, and a skeletal Class III pattern [19,22,26] (Figure 1). These complex manifestations tend to worsen over time, potentially compromising occlusal function, facial balance, and treatment outcomes [11]. Despite the clinical need, current evidence remains insufficient to support standardized orthodontic protocols for patients with moderate to severe forms of OI. As a result, early intervention is generally advised, guided by individualized treatment planning and a preference for techniques that impose minimal biomechanical stress on teeth and bone, given their inherent fragility [27,28,29].

### 4.2. Dentinogenesis Imperfecta: Orοfacial Manifestations and Orthodontic Treatment

Dentinogenesis imperfecta (DGI) is a hereditary disorder that may occur either concurrently with or independently of OI and can affect both primary and permanent dentition, with primary teeth being more severely impacted. The manifestation of DGI is highly prevalent in individuals with OI, and its severity correlates directly with the OI subtype. It occurs in approximately 80% of patients with type III OI, but it has also been reported in other types. The more severe the OI phenotype, the greater the frequency and severity of DGI. A strong association has been established between qualitatively abnormal type I collagen and DGI [12,14,18]. Clinically, affected teeth exhibit a yellow to bluish-brown discoloration due to abnormal dentine [30] and are prone to crumbling, as the enamel tends to detach from the underlying dentin. This enamel loss is not due to failure of the enamel–dentin junction, but rather to structural defects in the dentin itself [14,18]. In DGI, reduced collagen content within the dentin can alter the external contour of the teeth, particularly affecting crown morphology and dimensions, which may have implications for occlusion and orthodontic treatment planning [12].

Genetic differences have been identified between children exhibiting DGI exclusively in primary dentition and those affected in both primary and permanent dentitions. However, the underlying mechanisms determining why certain teeth are more severely affected than others remain incompletely understood. There is a higher frequency and greater severity of DGI in primary teeth and permanent lower incisors, and this is probably due to the reduced enamel thickness, which may enhance the translucency of the discolored and structurally compromised dentin [19,31]. In most patients, presenting DGI teeth are characterized by obliteration of the pulp chambers, short roots, and bulbous crowns [11].

A major consideration for orthodontists is that DGI can significantly impact the outcome of orthodontic treatment. Tooth fragility presents a challenge, as orthodontic forces applied to reposition teeth may lead to structural damage or fracture. Therefore, orthodontists must carefully modulate the applied forces to prevent iatrogenic complications, such as tooth or jaw fracture. Additionally, DGI affects the supporting periodontal structures, including the periodontal ligament and alveolar bone. These structures may be weaker or more susceptible to damage, necessitating a comprehensive assessment of dental integrity and stability before initiating orthodontic treatment. The anatomical variations associated with DGI—including alterations in tooth shape, size, and color—can further complicate tooth alignment and esthetic outcomes. As a result, adjunctive restorative procedures, such as resin composite bonding or veneers, may be required to enhance dental esthetics following orthodontic treatment. Furthermore, DGI can prolong orthodontic treatment duration. The compromised dentin structure necessitates gentler and more gradual tooth movement to minimize the risk of complications. Additionally, the need for extractions or restorative interventions to manage DGI-related dental defects can further extend the treatment timeline [14]. The application of clear-aligner therapy (CAT) to patients with OI and DGI could be more favorable than fixed appliances because aligners have greater elasticity and exert lighter forces [32].

In severe cases of OI, the extent of facial esthetic concerns, skeletal abnormalities, and occlusal dysfunction may necessitate orthognathic surgery as the most appropriate treatment approach. However, the combined orthodontic and surgical management of patients with OI, DGI, and skeletal Class III profile presents numerous challenges. Key considerations include bone and tooth fragility, an increased risk of secondary bleeding, and a predisposition to malignant hyperthermia postoperatively, which is described as one of the OI complications, due to instability of the autonomic nervous system. Additionally, intubation may be complicated due to macroglossia, short neck, and thoracic deformities commonly observed in these patients. Given the heightened fracture risk in OI, the surgical approach must be as minimally traumatic as possible. Patients should be fully informed of the risks and must provide informed consent prior to surgery [13,14]. Importantly, orthognathic surgery is typically performed in adults, as it requires the completion of skeletal development. However, in pediatric patients with OI, early orthodontic intervention is critical to mitigate the progression of skeletal Class III malocclusion, potentially reducing the need for future surgical correction, as mentioned before [11,24]. This highlights the importance of timely diagnosis and individualized treatment planning to optimize long-term functional and esthetic outcomes.

### 4.3. Other Forms of Primary Osteoporosis: Idiopathic Juvenile Osteoporosis

Several genetic disorders associated with bone fragility include Bruck syndrome, osteoporosis–pseudoglioma syndrome, idiopathic juvenile osteoporosis (IJO), and Cole–Carpenter syndrome [6]. Particular emphasis is placed on IJO, a rare and heterogeneous disorder with an unknown pathophysiology and no clear pattern of inheritance, which makes it a diagnosis of exclusion. Unlike most forms of genetic osteoporosis, IJO lacks a family history, extraskeletal manifestations, or evidence of growth disorder. The primary clinical features include bone pain (typically in the back, hips, and lower extremities), gait difficulties, vertebral compression fractures, and metaphyseal fractures. Symptoms typically manifest during school age and gradually regress over time. However, some complications, such as skeletal deformities or residual functional impairment, may persist. With advancements in genetic testing for osteoporosis, the number of cases classified as IJO has declined, and bone biopsy is now rarely required for diagnosis [4,6]. Currently, there is a lack of data in the literature regarding the dental and orthodontic conditions of patients with these genetic forms of osteoporosis. Future research is needed to assess whether these disorders have specific implications for occlusion, tooth development, or orthodontic management.

## 5. Pharmacological Therapy of Osteoporosis and Orthodontic Treatment

### 5.1. Bisphosphonates

Bisphosphonates are a synthetic class of drugs that act as stable analogs of pyrophosphoric acid. They exhibit a high affinity for calcium and can either accumulate in bone tissue, recirculate in the bloodstream, or be excreted in the urine. Due to their long half-life (up to ten years), bisphosphonates can remain in bone at high concentrations for extended periods [11,33]. Several types of bisphosphonates are available, and they can be administered either intravenously or orally. Notably, intravenous doses can be up to 12 times higher than oral doses [34].

Bisphosphonates have been used in the treatment of osteoporosis since the 1970s and remain the first-line therapy for pediatric osteoporosis, particularly in primary osteoporosis such as moderate to severe OI [7]. In most countries, their use in pediatric cases is considered off-label, requiring special regulatory approval. Their inhibitory effect on osteoclast activity leads to increased bone mass and mineralization, ultimately reducing fracture incidence. Numerous studies have demonstrated that bisphosphonate therapy effectively enhances bone density, alleviates pain, improves muscle strength, promotes growth, reduces skeletal deformities, and enhances mobility, thereby improving the overall quality of life in children with OI [15]. Currently, the most commonly used bisphosphonates for OI treatment include zoledronate, pamidronate, risedronate, and alendronate, with zoledronate and pamidronate being the preferred intravenous agents in pediatric patients [7]. Close dental monitoring is essential during bisphosphonate therapy to prevent potential adverse effects on dentition [11,15].

The administration of bisphosphonates in young children appears to influence dental morphology, particularly in the development of permanent teeth. In most case series, depending on disease severity, treatment initiation typically occurs around four years of age. However, in cases of severe phenotypes, some children begin therapy in infancy. Since permanent tooth formation occurs within the first four years of life, treatment during this period may interfere with odontogenesis. Malmgren et al. (2021) A significant association between early bisphosphonate therapy (before age 2) and an increased prevalence of tooth agenesis, particularly in children with OI types III and IV has been reported. However, there is a need for further research to clarify the full impact of bisphosphonate therapy on dental development, as variability in findings among different OI types suggests potential influencing factors that remain unclear [15].

An additional finding was the abnormal morphology of the premolars. In 32% of children treated with bisphosphonates before the age of two, premolars with an abnormal shape and dental intussusception (dens in dente and dens invaginatus) were found, while in 55% of these children, premolars presented with disorders such as microdontia and supernumerary occlusal cusps [15].

Several studies have concluded that bisphosphonates influence dental age and delay tooth eruption [7,11,20,35,36]. Their primary mechanism of action involves inhibiting osteoclast activity and bone resorption, processes essential for tooth eruption. As a result, bisphosphonates may contribute to delayed tooth eruption [35]. Importantly, findings from these studies indicate that bisphosphonate therapy perturbs dental age and delays tooth formation and eruption, particularly when administered before the age of two years [7].

It is well established that the rate of tooth movement varies among individuals, and patient responses to orthodontic treatment can differ significantly. Variability in the rate and extent of tooth movement may also be influenced by pharmacological agents and systemic factors. Studies have demonstrated that bisphosphonates can alter orthodontic tooth movement by affecting key cellular processes, ultimately inhibiting bone resorption. Additionally, some reports have identified an association between bisphosphonate use and serious jaw-related complications, primarily in adults (mentioned below) [31,34].

As previously mentioned, successful orthodontic treatment relies on osteoclast activity. Tooth movement requires functional osteoclasts to resorb bone in the compression zone and osteoblasts to form new bone in the tension zone. Bisphosphonates disrupt this remodeling cycle by inhibiting osteoclast function and reducing bone vascularity, which may impede orthodontic treatment by delaying or preventing tooth movement. Although direct evidence in humans is limited, an animal study in rats demonstrated that tooth movement decreased by 40% following three weeks of bisphosphonate administration [34]. These findings suggest a potential inhibitory effect on tooth movement that warrants further investigation in human populations.

Another bisphosphonate-related concern for orthodontists is the risk of bisphosphonate-induced jaw osteonecrosis (BIJON) [34]. Early reports raised concerns regarding the administration of bisphosphonates—particularly intravenous formulations—in patients with osteogenesis imperfecta due to the potential risk of developing osteonecrosis of the jaw following routine dental interventions, including simple tooth extractions [37,38]. Although its exact etiology remains uncertain, several theories have been proposed. In most cases, BIJON appears to be associated with impaired jawbone remodeling and abnormal wound healing. Inhibition of osteoclast function may contribute to delayed healing of local wounds, ultimately leading to jaw necrosis. Additionally, since orthodontic treatment stimulates alveolar bone remodeling, the risk of localized osteonecrosis may increase, particularly in patients wearing appliances that exert excessive pressure on the palate or those requiring surgical interventions, such as tooth extractions or mini-implant placement [34]. However, it is important to note that BIJON is a well-documented complication in adult patients receiving bisphosphonates, while no cases have been reported in children or adolescents. Although concerns have been raised regarding its potential impact on pediatric patients undergoing orthodontic treatment, current evidence does not support a direct association [39,40,41,42]. Further research is needed to determine its relevance in younger populations.

Given the established risk of osteonecrosis and impaired bone healing in patients receiving bisphosphonates, concerns have also been raised regarding their potential anti-angiogenic effects in the oral cavity. Specifically, high-dose bisphosphonate therapy may impair postoperative healing by inhibiting vascular endothelial growth factor (VEGF), a key regulator of angiogenesis. Zoledronic acid, in particular, has been shown to reduce the local bone blood supply, increasing the risk of ischemia and necrosis. Since the oral environment is highly susceptible to postoperative infections, proper surgical planning is essential. Any surgical procedure (e.g., premolar extraction for orthodontic purposes) should be performed as atraumatically as possible and not immediately after bisphosphonate administration [43].

Despite these potential risks, Rosen et al. (2011) concluded that a combination of orthodontic and orthognathic treatment remains feasible in OI patients receiving bisphosphonates, although the risk of complications is elevated [44]. Similarly, Munns et al. (2004) reported that in children with moderate to severe OI, bisphosphonate therapy was associated with delayed healing after osteotomy and recommended temporary suspension of therapy before and after surgery [45]. It should be emphasized that orthognathic surgery is primarily performed in adult patients, as it typically requires the completion of skeletal development. However, in exceptional and very rare cases, surgical intervention may be necessary during childhood to address severe skeletal discrepancies. Given this, early orthodontic intervention is crucial to prevent severe skeletal malformations before skeletal maturity, potentially reducing the need for surgical correction in adulthood.

Key recommendations for orthodontists managing patients receiving bisphosphonate therapy have been outlined [46]. These guidelines emphasize the importance of obtaining a comprehensive medical history, including bisphosphonate use, the treatment duration, dosage, and frequency of administration. Following this, a thorough risk–benefit assessment should be conducted to determine whether the patient is at high or low risk for complications, particularly osteonecrosis of the jaw. The level of risk is primarily associated with the route of bisphosphonate administration and the underlying condition being treated. For instance, intravenous bisphosphonate therapy for severe osteoporosis or cancer is significantly more likely to cause osteonecrosis and the suppression of osteoclastic activity, categorizing these patients as high-risk. In contrast, oral bisphosphonate therapy for osteopenia in adults carries a lower risk of developing osteonecrosis. Additionally, patients at high risk include those receiving bisphosphonates long-term, at high doses, or with frequent administration. While some high-risk patients may seek orthodontic treatment, it is advisable for orthodontists to postpone or avoid treatment in these individuals until the associated risks are minimized. For low-risk patients, if orthodontic treatment is deemed appropriate, treatment plans should be carefully evaluated and adjusted. In such cases, the following is recommended:

(1) Avoiding or minimizing surgical interventions and tooth extractions.
(2) Limiting orthodontic movements and compressive forces on teeth and surrounding tissues.
(3) Reducing the overall treatment duration whenever possible.

These modifications aim to mitigate potential complications while ensuring safe and effective orthodontic care for patients undergoing bisphosphonate therapy [46].

For patients receiving bisphosphonates who will eventually undergo orthodontic treatment, it is the orthodontist’s responsibility to consider the potential effects of these drugs on the alveolar bone throughout treatment. Even if bisphosphonate therapy is discontinued well before treatment initiation, the risk of complications remains uncertain, as the drug can persist in bone tissue for an extended period. Additionally, following the completion of orthodontic treatment, the final retention appliance should be entirely passive and subject to regular monitoring to minimize any potential risks [34].

Previous reports raised concerns regarding the use of rapid palatal expansion (RPE) in patients with moderate to severe OI, particularly when DGI is also present, citing risks related to skeletal fragility, poor sutural adaptability, and adverse root response to orthopedic forces [24]. However, more recent findings by Ierardo et al. (2015) demonstrated that young OI patients undergoing bisphosphonate therapy can exhibit favorable outcomes with Rapid Palatal Expansion (RPE), following standard force protocols and showing normal suture ossification and no complications at one-year follow-up [31]. These data suggest that, although caution is still warranted, RPE may be safely considered in selected cases, particularly those with milder forms of the disease.

Finally, it is crucial that patients receiving bisphosphonates and seeking orthodontic treatment, as well as their guardians (in the case of children and adolescents), fully understand that bisphosphonate therapy may slow orthodontic tooth movement and potentially impact treatment outcomes [34]. As previously mentioned, no cases of BRONJ have been reported in children in the international literature. This is likely due to the strict selection criteria for pediatric bisphosphonate administration and the use of relatively low doses in pediatric protocols.

Bisphosphonates have established their role as an effective and well-tolerated treatment for children with osteoporosis. However, regular dental and orthodontic monitoring is essential, as both the underlying disease and bisphosphonate therapy can contribute to dental and occlusal abnormalities [35]. Collaboration with the treating pediatrician is crucial to ensure optimal management, particularly when treatment modifications may be required—such as adjusting the regimen before tooth extractions or maxillofacial surgery.

### 5.2. Denosumab

Another therapeutic agent used in the management of osteoporosis is denosumab, a human monoclonal antibody that inhibits RANKL-RANK interaction and is approved for the treatment of osteoporosis. While its use in adults has shown promising results, its application in pediatric patients remains highly limited. There are several concerns regarding denosumab administration in children, including rebound hypercalcemia and vertebral fractures following treatment discontinuation. Consequently, references in literature remain scarce, as its use in pediatric populations is still under investigation. Further research is needed to determine both the appropriate dosing frequency for children and its potential systemic effects, particularly on dental and skeletal development [47,48].

## 6. Secondary Osteoporosis

Secondary osteoporosis can develop in children with chronic systemic diseases due to the direct effects of the underlying condition or its treatment. In these patients, increased bone fragility results from a complex interplay of multiple detrimental factors affecting bone metabolism and the functional musculoskeletal unit [2,49]. The most common causes of secondary osteoporosis are summarized in Table 1 [3] and include chronic inflammatory diseases, prolonged immobilization, eating disorders, malnutrition and/or malabsorption, altered pubertal development, neuromuscular disorders, solid organ transplantation, leukemia, and medication use (e.g., corticosteroids) [1,2,3,4,6,49,50].

These conditions are highly heterogeneous, differing in etiology, follow-up, and treatment protocols, making it challenging to establish universal guidelines in the literature [1,3,49].

Given the heterogeneity of conditions associated with secondary osteoporosis, the existing literature primarily focuses on how the underlying disease affects occlusion and orthodontic treatment, with limited reference to bone quality and quantity. Notably, there are no reports on the condition of the teeth when osteoporosis and/or bisphosphonate therapy coexist within the context of these diseases. As a result, the description of specific orthodontic anomalies and their management in patients with these conditions, without concomitant osteoporosis, falls beyond the scope of this targeted review. However, this gap in the literature highlights the need for future research, particularly to examine cases of secondary osteoporosis while considering the coexisting bone disorder and its treatment. Most importantly, orthodontists and dentists treating these groups of children must be aware of the potential risk of osteoporosis in their patients. A thorough understanding of their medication history, including bisphosphonates and other related treatments, is essential for optimizing orthodontic care.

Thus, in addition to pharmacological considerations, the influence of systemic conditions on craniofacial development and occlusion must also be considered. Moreover, systemic diseases associated with secondary osteoporosis, such as cerebral palsy and Duchenne muscular dystrophy, may predispose patients to orthodontic anomalies through multiple mechanisms [2]. Neuromuscular dysfunction may lead to alterations in orofacial muscular forces, resulting in asymmetrical craniofacial growth, anterior or posterior open bites, and crossbites [4,49,50]. Additionally, prolonged immobilization and reduced mechanical loading negatively affect bone modeling and remodeling during critical periods of skeletal development, potentially exacerbating the severity of malocclusions. These factors should be carefully considered during orthodontic treatment planning in this patient population [1,3,4,6,49,50].

## 7. Clinical Considerations and Research Gaps

Given the rarity and heterogeneity of pediatric osteoporosis, current research remains limited and fragmented, underscoring the necessity for a cautious, individualized, and multidisciplinary approach to orthodontic management to minimize iatrogenic risks and optimize clinical outcomes [15,27,28,35]. To address these challenges, effective orthodontic management requires careful adaptation to each patient’s needs, with close collaboration among dental, medical, and surgical teams [14,27]. Although bisphosphonate therapy contributes to improved bone density, it may impair bone remodeling and slow orthodontic tooth movement [15,31,34], necessitating thorough risk assessment prior to treatment [11]. The use of low-force mechanics, the minimization of invasive procedures, and consistent communication with the patient’s healthcare providers are crucial to mitigating complications. Furthermore, early diagnosis and comprehensive counseling of patients and caregivers are key to maximizing functional and esthetic outcomes over the long term. Early orthodontic intervention is generally recommended, guided by individualized treatment planning and a preference for techniques that impose minimal biomechanical stress on teeth and bone, given their inherent fragility [11,27,29]. It is generally accepted that patients with type 1 OI and mild DGI who present with moderate malocclusions may be successfully managed using conventional orthodontic techniques [11,23,24].

Most current evidence on bisphosphonate effects in dentistry and orthodontics comes from animal studies; caution is warranted when extrapolating these results to human populations. Fundamental differences in bone metabolism, pharmacokinetics, and growth dynamics between animal models and humans may limit the relevance of these findings to clinical practice [34]. Consequently, there is a pressing need for rigorously designed longitudinal clinical studies in pediatric populations to elucidate the specific effects of bisphosphonate therapy on craniofacial growth, odontogenesis, and orthodontic treatment responses.

The limitations of this narrative literature review include the low number of high-quality clinical studies, the reliance on a single database for the literature search, and the exclusion of non-English articles due to language proficiency constraints. Nevertheless, efforts were made to ensure methodological transparency through strict inclusion and exclusion criteria and a systematic screening process, thereby minimizing selection bias

## 8. Conclusions

This narrative literature review summarizes reported findings regarding dental and orthodontic challenges in pediatric patients with osteoporosis. To date, most studies focus on primary pediatric osteoporosis, particularly osteogenesis imperfecta (OI), where bisphosphonate therapy is commonly used. According to the current literature, these patients tend to present with more complex dental and skeletal anomalies, such as skeletal Class III profiles, dentinogenesis imperfecta (DGI), tooth agenesis, delayed tooth eruption, and anterior or posterior crossbites. The severity and combination of these manifestations appear to correlate with the degree of bone involvement.

Future research should aim to document the natural history and progression of orthodontic anomalies in pediatric patients with osteoporosis and to evaluate the long-term safety and efficacy of orthodontic interventions in this vulnerable population.

## Figures and Tables

**Table 1:** Causes of secondary osteoporosis [3].

Category	Example
Chronic inflammatory disorders	Autoimmune rheumatoses
Inflammatory bowel disease	Crohn’s disease
Malabsorption syndrome	Cystic fibrosis, Coeliac disease
Chronic immobilization	Cerebral palsy, Duchenne’s muscular dystrophy
Epidermolysis bullosa	-
Endocrine disorders	Amenorrhea, hyperthyroidism, hypogonadism
Cancer and treatments with adverse effects on bone health	Chronic corticosteroid therapy, chemotherapy
Hematological disorders	Thalassemia
Genetic disorders with bone complications	-

## Data Availability

Not applicable.

## Abbreviations

The following abbreviations are used in this manuscript:

BIJON	Bisphosphonate-induced jaw osteonecrosis.
DGI	Dentinogenesis imperfecta.
IJO	Idiopathic juvenile osteoporosis.
OI	Osteogenesis imperfecta.
RPE	Rapid palatal expansion.
TMJ	Temporomandibular joint.
VEGF	Vascular endothelial growth factor.
